# Crosstalk between TLR8 and RIG-I-like receptors enhances antiviral immune responses

**DOI:** 10.3389/fmed.2023.1146457

**Published:** 2023-05-16

**Authors:** Killian E. Vlaming, Kelly van Wijnbergen, Tanja M. Kaptein, Monique Nijhuis, Neeltje J. Kootstra, Godelieve J. de Bree, Teunis B. Geijtenbeek

**Affiliations:** ^1^Department of Experimental Immunology, Amsterdam UMC Location University of Amsterdam, Amsterdam, Netherlands; ^2^Amsterdam Institute for Infection and Immunity, Amsterdam, Netherlands; ^3^Translational Virology, Department of Medical Microbiology, University Medical Center Utrecht, Utrecht, Netherlands; ^4^Department of Internal Medicine, Amsterdam UMC Location University of Amsterdam, Amsterdam, Netherlands

**Keywords:** toll like 7/8 receptors, RIG-I like receptors (RLRs), immune activation, antiviral responses, crosstalk

## Abstract

**Background:**

Toll-like receptor (TLR) agonists have been investigated due to their potential dual effects as latency reverting agents and immune modulatory compounds in people living with HIV (PLWH). Here, we investigated whether co-stimulation of TLR7/8 agonists with RIG-I-like receptor (RLR) agonists enhances antiviral immunity.

**Methods:**

Peripheral blood mononuclear cells (PBMCs) and monocyte-derived dendritic cells (DCs) were incubated with TLR and RLR-agonists for 24 h and innate and adaptive immune responses were determined (maturation markers, cytokines in supernatant, ISG expression).

**Results:**

Both TLR7 and TLR8 agonists induced pro-inflammatory cytokines in DCs as well as PBMCs. TLR8 agonists were more potent in inducing cytokine responses and had a stronger effect on DC-induced immunity. Notably, while all compounds induced IL-12p70, co-stimulation with TLR8 agonists and RLR agonist polyI: C induced significantly higher levels of IL-12p70 in PBMCs. Moreover, crosstalk between TLR8 and RLR agonists induced a strong type I Interferon (IFN) response as different antiviral IFN-stimulated genes were upregulated by the combination compared to the agonists alone.

**Conclusion:**

Our data strongly suggest that TLR crosstalk with RLRs leads to strong antiviral immunity as shown by induction of IL-12 and type I IFN responses in contrast to TLRs alone. Thus, co-stimulation of TLRs and RLRs might be a powerful strategy to induce reactivation of latent reservoir as well as antiviral immunity that eliminates the reactivated cells.

## Introduction

The worldwide pandemic of HIV still poses significant challenges to public health. While antiretroviral therapy (ART) has significantly improved the survival chances and quality of life for those undergoing HIV infection, it has so far failed to achieve cure (1, 2). Cure can be achieved in various ways with a specific focus on eliminating the HIV reservoir, a cellular reservoir that contains latently infected cells, which become virus producers upon removal of antiretroviral therapy (3–5). So far the only successful documented HIV cure have been achieved by bone marrow transplantation with donor CCR5 delta32 cells (1). A proposed strategy for cure involves a combined approach where the HIV reservoir is eliminated by either reactivation and subsequent elimination by different approaches including HIV-1-specific immunity (6). HIV-1-specific immunity in PLWH is insufficient to control in most cases viral replication and it is becoming clear that therapies that enhance HIV-1-specific immunity are important to curb viral replication and eliminate reactivated reservoirs in HIV cure therapies. Moreover, there are several cases of people living with HIV (PLWH) that control their virus without antiretroviral therapy most likely *via* a very efficient and specific HIV-1-targeted immune responses (7, 8). The immune control displayed by this group is highly interesting and although underlying immune responses remain unclear, these studies underscore the importance of immunity in controlling HIV-1.

Current studies have shown that latency reversal agents (LRAs) are able to induce HIV RNA transcription and virion production *in vivo* but even in combination with immunotherapeutics fail to decrease HIV reservoir (9–13). This could be due to poor antigen activity of LRAs or insufficient immune activation or efficacy of the immunotherapeutics (12). Toll-like receptor (TLR) agonists are being studied most widely as adjuvants to enhance anti-HIV-1 immunity but with limited success (6, 9, 14–16).

TLRs are pattern recognition receptors (PRRs) expressed by immune cells in particular antigen presenting cells. These PRRs are type-I transmembrane proteins and recognize pathogen-associated molecular patterns (PAMPs). TLRs are expressed on the cellular surface or in endosomal vesicles where they recognize cell-wall components such as lipoproteins, proteins and lipids, or nucleic acids, respectively. TLR activation leads to the induction of transcription factors, in particular NFκB and different Interferon Regulatory Factors (IRFs), that combined induce specific immune responses (17). The adjuvant capacity of TLR7 and TLR8 agonists has been studied in HIV cure (9, 14–16). TLR7 and TLR8 are both located endosomally and are able to detect single stranded RNA (ssRNA) including HIV-1 ssRNA (18, 19).

Several agonists of TLR7 and TLR8 are currently under investigation for *in vivo* induction of immune responses (9, 14, 20–23). TLR7 agonists have been previously investigated for their potency in contributing to HIV cure. Vesatolimod (GS-9620), a selective TLR7 agonist, was able to induce antiviral responses in PLWH undergoing ART, as measured by IP-10, IL-1RA and ITAC levels in serum (9). Furthermore, in non-human primates it was observed that the TLR7 agonist could delay viral rebound after cessation of ART, thus providing a possible first step to eventual cure (24). TLR8 agonists have been less investigated so far (14, 15). These studies show that TLR7 and 8 agonists might have the potential to induce antiviral immunity but their signaling is limited to specific immune activation programs that might not be sufficient to induce effective immunity to HIV-1 during reactivation. There are other PRR families such as the family of cytosolic RIG-I like receptors (RLR) RIG-I and MDA5 that sense double stranded RNA structures, replication intermediates for RNA viruses (25). RIG-I/MDA5 triggering during virus replication leads to MAVS dependent activation of NFkB and IRF3 and subsequent cytokine and type I IFN responses (25, 26).

Notably, recent studies suggest that crosstalk between signaling by TLRs and other PRRs including C-type lectin receptors and RLRs modulate and enhance adaptive immunity (27–34). However, it remains unclear whether TLR7/8 induced immunity can be enhanced by co-stimulation of the RLR signaling axis. Therefore, we investigated TLR7/8-induced immune responses induced by DCs and PBMCs and how crosstalk with RLR signaling affects the adaptive immune responses. We investigated different TLR7 and TLR8 agonists and TLR8 stimulation was more effective than TLR7 in induction of pro-inflammatory cytokines. Notably, co-stimulation of TLR8 with RIG-I/MDA5 agonists enhanced induction of pro-inflammatory cytokines as well as type I IFN responses. These data suggest that co-stimulation of TLR8 with RLR agonists enhance antiviral immunity and could therefore be used in HIV cure strategies.

## Methods

### Ethics statement

This study was performed according to the University Medical Center Amsterdam, location AMC, Medical Ethics committee guidelines and according to the Declaration of Helsinki.

### Cell culture

PBMCs were obtained from buffy-coats of healthy donors (Sanquin). PBMCs were isolated by a Lymphoprep (Axis-shield) gradient. PBMCs were cultured in RPMI medium enriched with 10% FCS (Biological Industries), 10 IU/mL penicillin (Thermo Fisher), 10 mg/mL streptomycin (Thermo Fisher), 2 mM L-glutamine (Lonza) and 10 IU/mL IL-2 (Invivogen).

DCs were generated from PBMCs isolated from buffy-coats of healthy donors. Monocytes were isolated by a Percoll (Amersham biosciences) gradient step. Immature monocyte-derived DCs were cultured for 6–7 days from monocytes in the presence of RPMI medium enriched with 10% FCS (Biological Industries), 10 IU/mL penicillin (Thermo Fisher), 10 mg/mL streptomycin (Thermo Fisher), 2 mM L-glutamine (Lonza), IL-4 (500 U/mL, bioscource) and GM-CSF (800 U/mL, invivogen). DCs were defined by high expression of CD209 and CD11c, and low expression of CD14.

PBMCs and DCs were played on round-bottom culture plates (150.000 PBMCs per condition, 100.000 DCs per condition). Cells were stimulated for 24 h in the presence of TLR agonists R837 (selective TLR7), R848 (TLR7 & TLR8) and TL8-052 (selective TLR8) as well as TLR-agonists of clinical interest: two selective TLR7 agonists; Vesatolimod (GS-9620), Gardiquimod. TLR7 and TLR8 agonist Telratolimod (3 M-052) and two selective TLR8 agonists; Selgantolimod (GS-9866, selective TLR8 agonist) and Motolimod (VTX-2337) (9, 14, 20–23). Stimulations for ELISA were performed for three biological donors, each biological donor supplied material for three technical triplicates. Technical triplicates were included for analysis but grouped together when comparing effects between donors.

Cells for RT-PCR were plated in round-bottom culture plates (100.000 DCs) per condition. Three biological donors were included and seeded in mono. Donors supplied cells for both Elisa and RT-PCR experiments.

### Stimuli

R837 (selective TLR7 agonist), R848 (TLR7 & TLR8 agonist) and TL8-052 (selective TLR8 agonist) were acquired from Invivogen and stored at −20°C. TLR-agonist R837 was used at a concentration of 5 μg/mL. R848 at 10 μg/mL and TL8-052 at 5 μg/mL.

Vesatolimod, Gardiquimod, Telratolimod, Selgantolimod and Motolimod were acquired from a commercial distributor (MedChemExpress) and stored as per the manufacturer’s instructions. Concentrations were used in increasing concentration to establish working concentrations as follows: TLR-agonist GS-9620/Vesatolimod, 0.1 μM, 1 μM, 3 μM, 10 μM, 20 μM, 30 μM, 60 μM (selective TLR7 agonist), Gardiquimod, 0.1 μM, 1 μM, 3 μM, 10 μM, 20 μM, 30 μM (selective TLR7 agonist), 3 M-052/Telratolimod, 0.1 μM, 1 μM, 3 μM, 10 μM, 20 μM, 30 μM (TLR7 & TLR8 agonist), GS-9688/Selgantolimod, 0.1 μM, 1 μM, 3 μM, 10 μM, 20 μM, 30 μM (selective TLR8 agonist), MTX-1337/Motolimod, 0.1 μM, 1 μM, 3 μM, 10 μM, 20 μM, 30 μM (selective TLR8 agonist).

PolyIC-lyovec (LMW) was acquired from Invivogen and dissolved in LAL-water as per the manufacturer’s instructions and used at a concentration of 2 μg/mL. Salmonella Lipopolysaccharide (LPS) was used as positive controls at a concentration of 10 ng/mL.

### Flow cytometry

DCs were stimulated for 24 h and stained with PE-conjugated anti-CD80 (1:12.5, 557,227, BD pharmingen), allophycocyanin-conjugated CD83 (1:25, 551,073, BD Pharmingen), FITC-conjugated anti-CD86 (1,25, 555,657, BD Pharmingen). *The gating strategy used and histograms of a representative donor are displayed in* Supplementary Figure S1. Flow cytometry was performed on the FACS Canto II (BD Biosciences) and analysed *via* FlowJo software (v10.8.2).

### Elisa

Supernatant of DC or PBMCs were harvested at select timepoints after stimulation. Subsequently secretion of IL-6, IL-10 and IL-12p70 proteins were measured by ELISA as described by the manufacturer (eBiosciences). OD450nm values were measured using BioTek synergy HT.

### Quantitative real-time PCR

mRNA was acquired following lysis of cellular material at set time points, it was transcribed to cDNA using a reverse transcriptase kit (Promega). Quantitative real-time PCR was performed on an ABI 7500 Fast real-time PCR detection system from Applied Biosystems using SYBR green (Thermo fisher).

Expressions of genes of interests were normalized to a household gene (GAPDH). The formula used was Nt = 2^Ct(GAPDH) − Ct(target)^. Expression was subsequently normalized to expression of the TLR-stimulus at *T* = 8 h.

### Statistical analysis

Statistical analysis of obtained data was performed using Graphpad Prism 9 (Graphpad Software Inc).

Two-way ANOVA tests were performed to compare unpaired grouped data between donors (IL-6, IL-10, IL-12p70 Elisa). One-way ANOVA tests were performed for unpaired observations between donors (RT-PCR). A two-tailed student’s *t*-test was used for paired observations and descriptive statistics were used to calculate time-courses. Statistical significance was set at *p < 0.05, **p < 0.01, ***p < 0.001, ****p < 0.0001.

## Results

### TLR8 but not TRL7 stimulation induced dendritic cell maturation and cytokines

**Figure 1:**
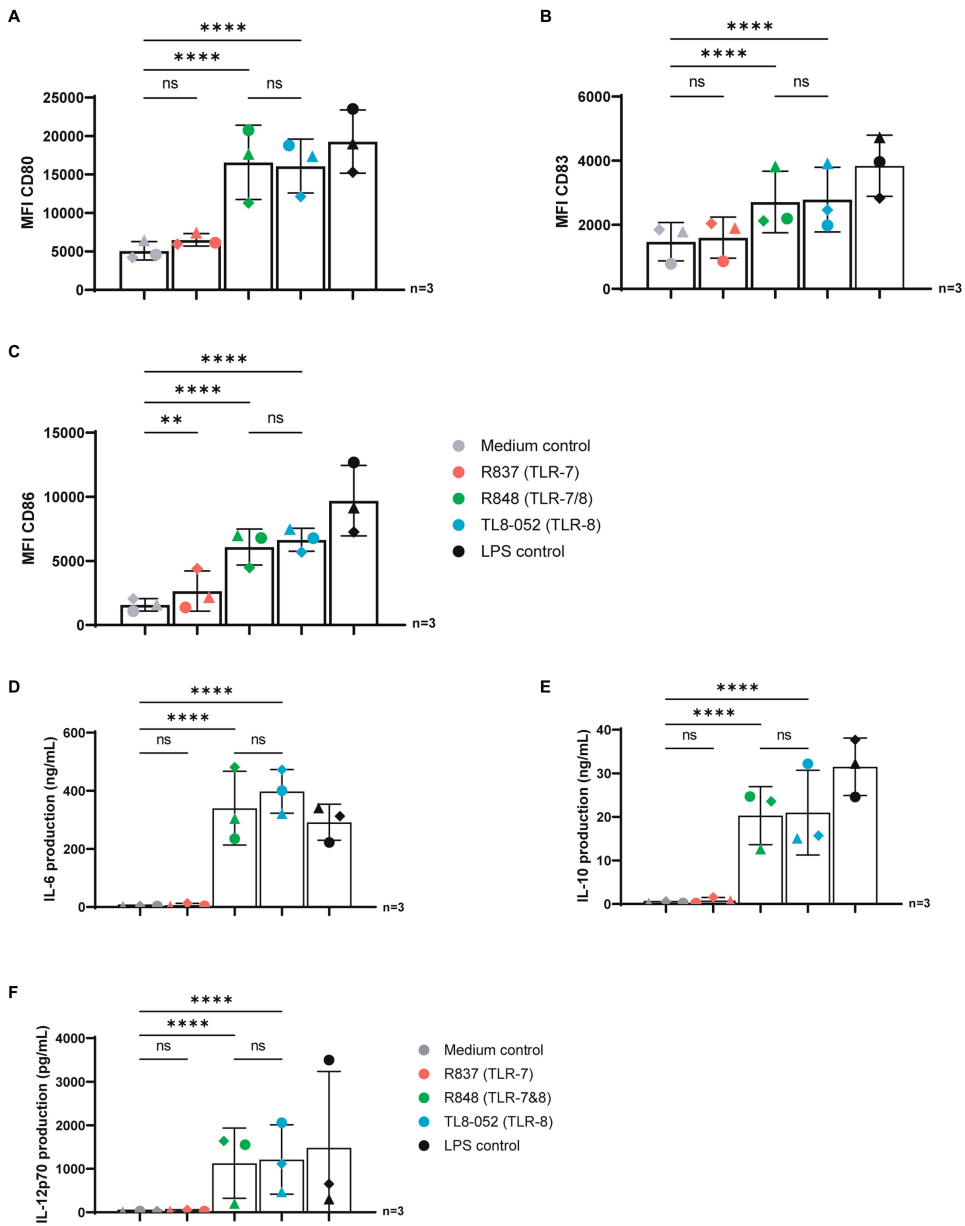
TLR-8 but not TRL7 stimulation induced maturation and cytokine production in DCs. **(A–C)** DCs were stimulated for 24 h with different TLR agonists and expression of CD80, CD83 and CD86 was measured by flow cytometry. **(D–F)** Supernatant of DCs were harvested after 24 h stimulation. Secretion of IL-6, IL-10 and IL-12p70 proteins were measured by ELISA as described by the manufacturer (eBiosciences). OD450nm values were measured using BioTek synergy HT. Statistical analysis of obtained data was performed using Graphpad Prism 9 (Graphpad Software Inc). One-way ANOVA tests were performed for unpaired observations between donors (CD-80, CD-83, CD-86 expression). Two-way ANOVA tests were perfomed to compare unpaired grouped data between donors (IL-6, IL-10, IL-12p70 Elisa). Statistical significance was set at ns *p* > 0.05, ^*^p < 0.05, ^**^p < 0.01, ^***^p < 0.001, ^****^p < 0.0001.

We set out to assess the potency of three known TLR agonists to activate DCs. We stimulated human-monocyte derived DCs with R837 (selective TLR7 agonist), R848 (TLR7 & TLR8 agonist) and TL8-052 (selective TLR8 agonist) for 24 h and maturation was determined by measuring expression of co-stimulatory molecules by flow cytometry. Stimulation of DCs with TLR7-agonist R837 did not induce expression of CD80 and CD83 as compared to unstimulated DCs (Figures 1A–C; Supplementary Figure S1). CD86 was increased to a low extent upon stimulation with R837 (Figure 1C). In contrast to stimulation with TLR7 agonists R837, stimulation with TLR7/8 and TLR8 agonists R848 and TL8-052 induced DC maturation as observed by strong upregulation of CD80, CD83 and CD86, compared to the medium control (p < 0.0001) and to a similar extent as the positive control LPS that triggers TLR4 (Figures 1A–C; Supplementary Figure S1). Subsequently, we investigated cytokine production by DCs upon 24 h stimulation with different agonists (Figures 1D–F). Stimulation with R837 (TLR7) did not induce IL-6, IL-10 or IL-12p70 (Figures 1D–F) as compared to the medium control. In contrast, R848 (TLR7&8) and TL8-052 (TLR8) induced expression of IL-6, IL-10, and IL-12p70 to a similar extent as the positive control LPS, compared to the medium control (*p* < 0.0001) (Figures 1D–F). These data suggest that TLR8 agonists are more potent than TLR7 agonists in inducing DC-mediated immune responses.

TLR7 and TLR8 agonists induced immune activation in peripheral blood immune cells.

**Figure 2:**
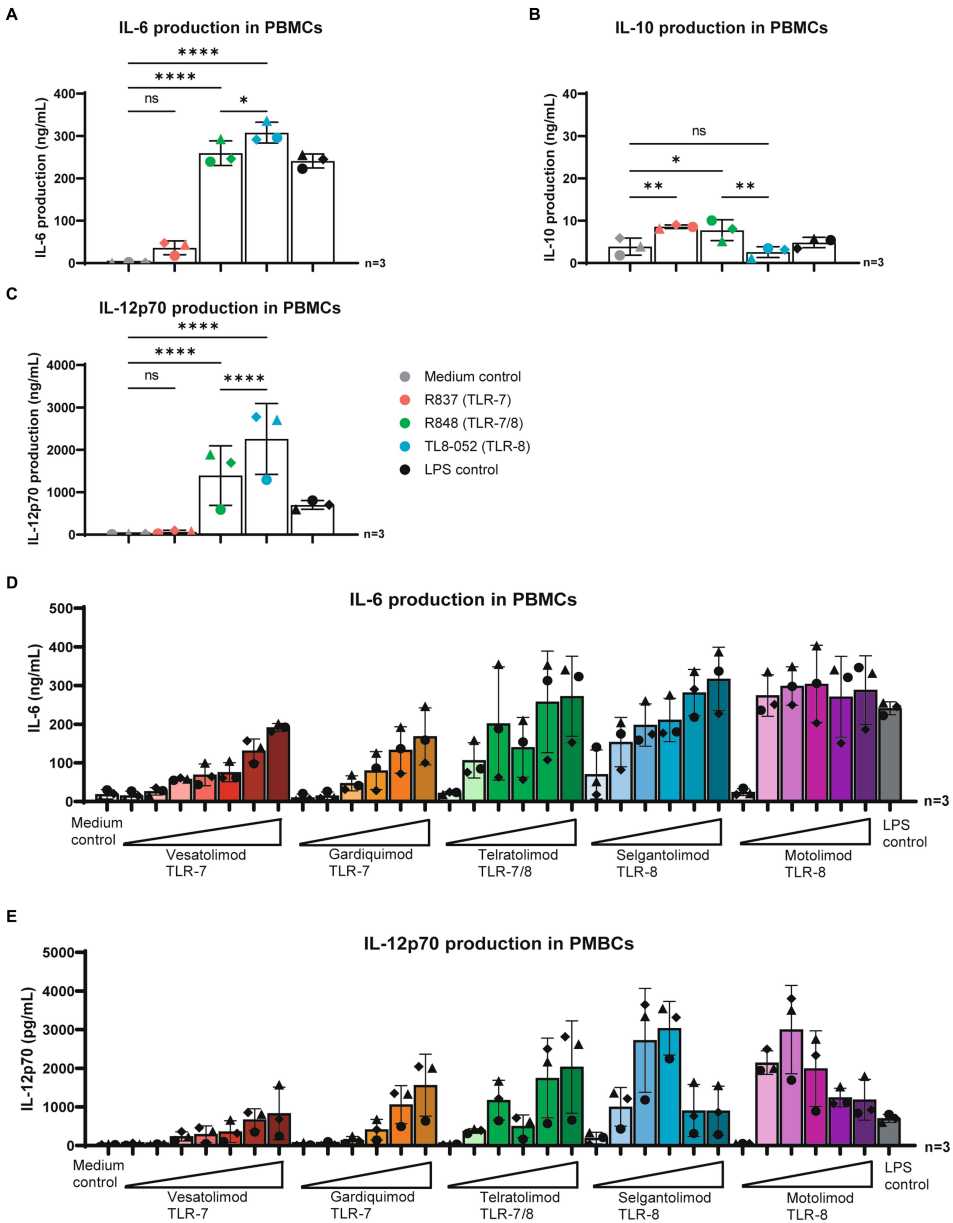
TLR-7 and TLR-8 agonists induced immune activation in PBMCs. **(A–C)** PBMCs were stimulated for 24 h in the presence of different TLR agonists. Supernatant was harvested after 24 h stimulation, secretion of IL-6, IL-10 and IL-12p70 proteins were measured by ELISA as described by the manufacturer (eBiosciences). OD450nm values were measured using BioTek synergy HT. **(D,E)** PBMCs stimulated for 24 h in the presence various TLR-agonist in varying concentrations. Supernatant was harvested after 24 h stimulation. Secretion of IL-6 and IL-12p70 proteins were measured by ELISA as described by the manufacturer (eBiosciences). OD450nm values were measured using BioTek synergy HT. Obtained values were normalized to values obtained from LPS stimulation to allow for better comparison between donors. Two-way ANOVA tests were perfomed to compare unpaired grouped data between donors (IL-6, IL-10, IL-12p70 Elisa). Statistical analysis of obtained data was performed using Graphpad Prism 9 (Graphpad Software Inc). Statistical significance was set at ns *p* > 0.05, ^*^p < 0.05, ^**^p < 0.01, ^***^p < 0.001, ^****^p < 0.0001.

Next, we investigated the immune responses induced by TLR7 and TLR8 agonists in peripheral blood mononuclear cells (PBMCs). PBMCs were stimulated with TLR agonists R837 (TLR7), R848 (TLR7&8) and TL8-052 (TLR8) for 24 h and cytokines were measured in supernatant by ELISA. Stimulation with R837 (TLR7) induced low levels of IL-6 and IL-10 but no IL-12p70 in PBMCs (Figures 2A–C). As observed with DCs, stimulation of PBMCs with R848 (TLR7&8) and TL8-052 (TLR8) induced IL-6 similar as observed for the LPS control. R837 and R848 induced a significant but minor increase in IL-10 production. Moreover, high levels of IL-12p70 were observed following stimulation with R848 (TLR7/8) and TL8-052 (TLR8) (Figure 2C).

Subsequently, PBMCs were stimulated with five TLR-agonists undergoing clinical evaluation (9, 14, 20–23) and cytokines were measured. Neither of the stimuli induced an IL-10 responses (data not shown). Stimulation with TLR7-agonists Vesatolimod and Gardiquimod induced IL-6 and IL-12p70 secretion in a dose dependent manner (Figures 2D,E). TLR 7/8 agonist Telratolimod similarly induced a dose dependent increase in IL-6 and IL-12p70 (Figures 2D,E). TLR8 agonists Selgantolimod and Motolimod, induced high levels of cytokines at low concentrations. IL-6 production followed a dose dependent concentration curve for Selgantolimod and reached a plateau for Motolimod at 1 μM, with reaching a plateau at higher concentrations. IL-12p70 production peaked at low concentrations for both Selgantolimod and Motolimod. These data suggest that both TLR7 and TLR8 agonists induce proinflammatory cytokines in PBMCs and potency depends on the agonist even though TLR8 agonists seem more potent.

### RLR crosstalk with TLR7/8 and TLR8 induced IL-12p70 but restricted IL-6 expression in PBMCs

**Figure 3:**
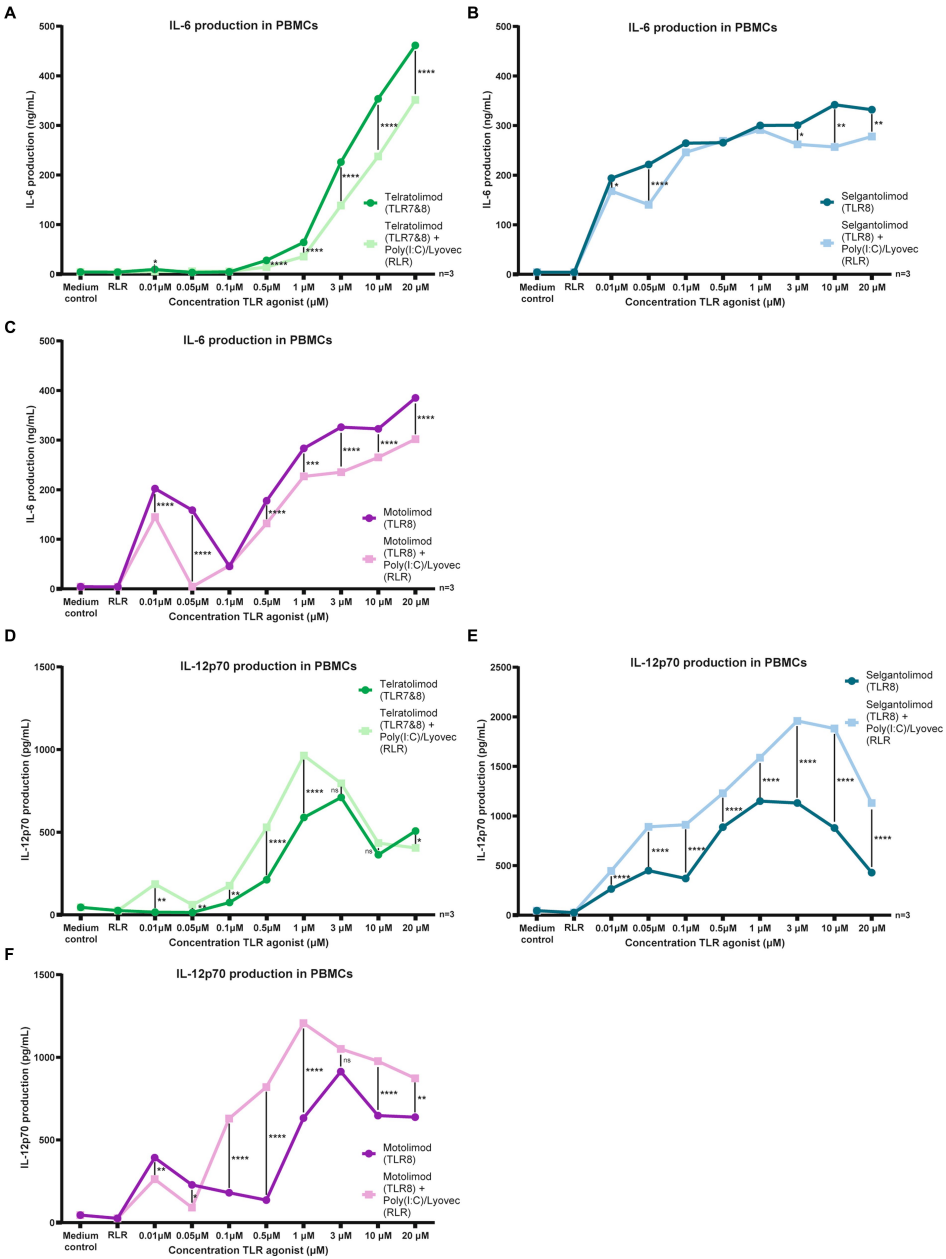
Co-stimulation of TLR-8 and RLR restricts IL-6 and induces IL-12 secretion in PBMCs. **(A–F)** PBMCs were stimulated for 24 h in the presence of RLR agonist and different TLR agonists at various concentrations. Supernatant was harvested after 24 h stimulation. IL-6 and IL-12p70 proteins were measured by ELISA as described by the manufacturer (eBiosciences). Datapoints shown are averaged data from three individual donors. OD450nm values were measured using BioTek synergy HT. Two-way ANOVA tests were perfomed to compare unpaired grouped data between donors (IL-6, IL-10, IL-12p70 Elisa). Statistical analysis of obtained data was performed using Graphpad Prism 9 (Graphpad Software Inc). Statistical significance was set at ns *p* > 0.05, ^*^p < 0.05, ^**^p < 0.01, ^***^p < 0.001, ^****^p < 0.0001.

To elucidate effects of co-stimulation between TLR7/8 (Telratolimod) or TLR8 agonists (Selgantolimod and Motolimod) and the RLR agonist Poly (I:C) (LMW)/Lyovec on cytokine production in PBMCs we co-stimulated PBMCs in the presence of TLR and RLR agonists at various concentrations. The RLR-agonist Poly (I:C) (LMW)/Lyovec alone did not had no effect on IL-6 production (Figures 3A–C; Supplementary Figures S4A–C). No effect was observed on IL-12p70 secretion either (Figures 3D–F; Supplementary Figures S4D–F). To elucidate compound specific effects of Poly (I:C) (LMW)/Lyovec experiments were replicated with Poly (I:C) (HMW)/Lyovec, which showed similar results to the LMW variant (data not shown).

Stimulation using Telratolimod (TLR 7&8) showed a dose dependent increase of IL-6 production, but, notably, co-stimulating with Poly(I:C) (LMW)/Lyovec decreased IL-6 production (Figure 3A; Supplementary Figure S4A). This decrease was also observed at multiple concentrations for both Selgantolimod (TLR8) and Motolimod (TLR8) (Figures 3B,C; Supplementary Figures S4B,C).

In contrast to the effects observed on IL-6 production, co-stimulation with Poly(I:C) (LMW)/Lyovec increased IL-12p70 production. An interesting drop in stimulatory activity was observed for both Motolimod at 0.1 μM, likely caused by large donor variation at lower concentrations (Supplementary Figure S4C).

For both Telratolimod (TLR7&8) and Motolimod (TLR8) an increase in IL-12p70 secretion was observed at multiple concentrations on a wide range (Figures 3D,F; Supplementary Figures S4D,F). For Selgantolimod (TLR8) this increase was observed at every concentration tested (Figure 3E; Supplementary Figure S4E). An interesting drop in stimulatory activity was observed for both Telratolimod and Motolimod at 0.05 μM, likely caused by large donor variation at lower concentrations (Supplementary Figure S4).

### Co-stimulation of TLR8 and RLR induced type I IFN responses

**Figure 4:**
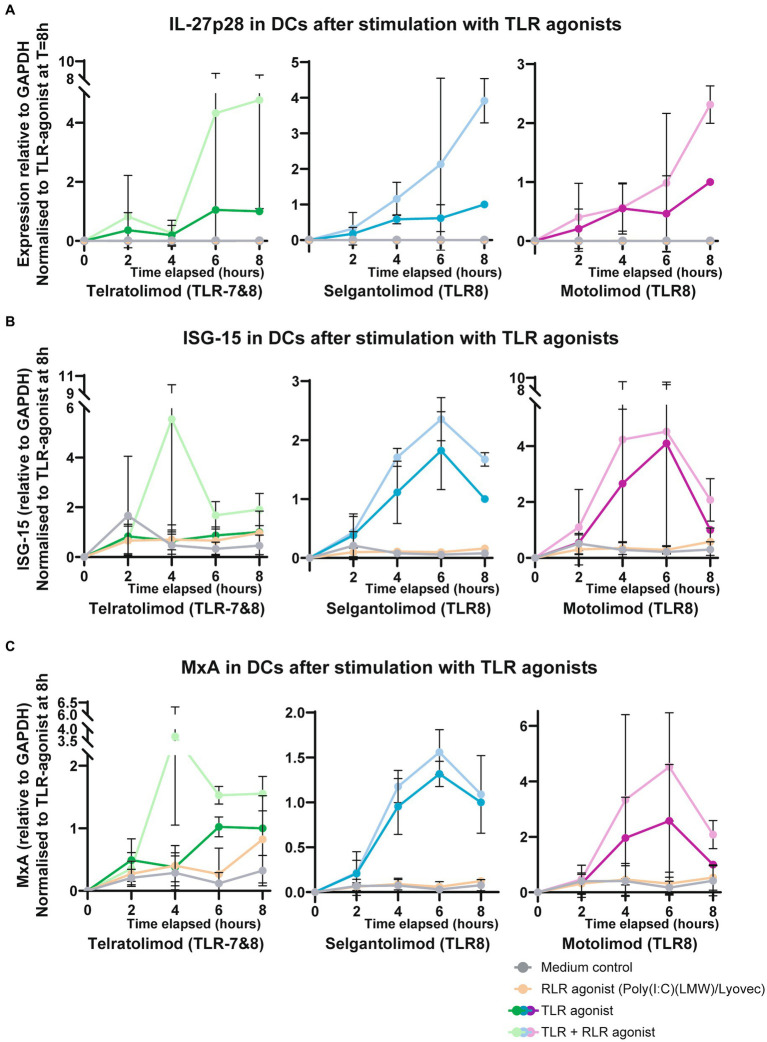
Co-stimulation of TLR-8 and RLR induced potent antiviral cytokines in DCs. DCs were stimulated with RLR and different TLR-agonists. mRNA was extracted to investigate genes of interest. Quantitative real-time PCR was performed on ABI-7500 Fast real-time PCR detection system (Applied Biosystems) using SYBR green (Thermo Fisher). **(A–C)** Expression of genes of interest; IL-27A, ISG-15. MxA, were normalized to a household gene (GAPDH). The formula used was Nt = 2Ct(GAPDH) − Ct(target). Expression was subsequently normalized to expression of the TLR-stimulus at *T* = 8 h. Statistical analysis of obtained data was performed using Graphpad Prism 9 (Graphpad Software Inc). One-way ANOVA tests were performed for unpaired observations between donors (CD-80, CD-83, CD-86 expression). Statistical significance was set at ns *p* > 0.05, ^*^p < 0.05, ^**^p < 0.01, ^***^p < 0.001, ^****^p < 0.0001.

**Figure 5:**
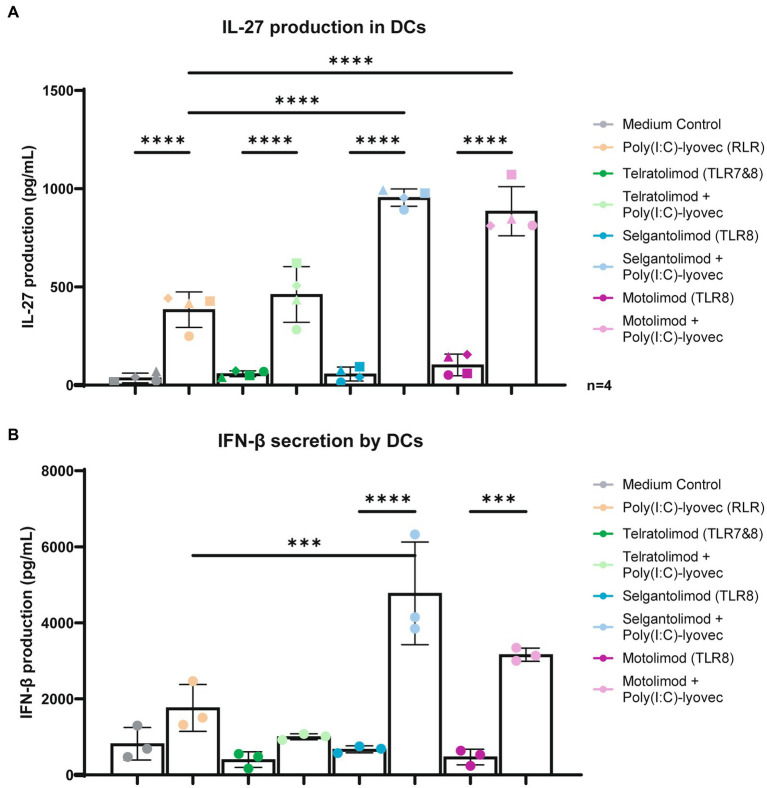
Co-stimulation of TLR-8 and RLR induce IL-27 and IFN-β secretion in DCs. **(A)** DCs were stimulated for 24 h in the presence of RLR agonist and different TLR agonists at various concentrations. Supernatant was harvested after 24 h stimulation. IL-27 proteins were measured by ELISA as described by the manufacturer (U-CyTech biosciences). **(B)** DCs were stimulated for 6 h in the presence of RLR agonist and different TLR agonists at various concentrations. Supernatant was harvested after 6 h stimulation. IFN-*β* proteins were measured by ELISA as described by the manufacturer (R&D systems). Technical triplicate values from a representative donor have been added into a Figure of a representative donor. OD450nm values were measured using BioTek synergy HT. Two-way and one-way ANOVA tests were perfomed to compare unpaired grouped data between donors. Statistical analysis of obtained data was performed using Graphpad Prism 9 (Graphpad Software Inc). Statistical significance was set at ns *p* > 0.05, ^*^p < 0.05, ^**^p < 0.01, ^***^p < 0.001, ^****^p < 0.0001.

IFN-stimulated gene IL-27 has been shown to be important in the activation of CD8 T cell responses as well as in the induction of follicular T helper cells (27, 35–37). Moreover, other ISGs have been suggested to be important in antiviral immunity such as ISG-15, MxA, IP10 and A3G (38–42). Here we investigated whether co-stimulation with RLR agonist Poly(I:C)/Lyovec affects the immune responses induced by TLR7/8 and TLR8 agonists. DCs were stimulated with Telratolimod (TLR7/8) and TLR8 agonists Selgantolimod and Motolimod alone or in combination with RLR-agonist Poly(I:C) (LMW)/Lyovec and specific IFN-stimulated genes were measured. Although stimulation with either RLR, TLR8 or TLR7/8 agonists alone induced low levels of mRNA levels of ISG IL-27A, co-stimulations of TLR8 agonists with Poly(I:C) (LMW)/Lyovec enhanced expression of IL-27A in time (Figure 4A). We did not observe a significant increase when combining TLR7/8 Telratolimod with Poly(I:C) (LMW)/Lyovec. Notably, crosstalk between Poly(I:C) (LMW)/Lyovec and TLR8 agonists Selgantolimod and Motolimod enhanced IL-27 protein secretion compared to either stimulus alone (Figure 5A). Crosstalk of TLR8 agonist with Poly(I:C) (LMW)/Lyovec resulted in a two-fold increase of IL-27 compared compared to Poly(I:C) (LMW)/Lyovec alone. We did not observe any enhancement with TLR7 agonists compared Poly(I:C) (LMW)/Lyovec alone (data not shown).

Moreover, crosstalk between the TLR8 agonists and Poly(I:C) (LMW)/Lyovec increased protein secretion IFN-β with a two to nine fold increase compared to the medium control (Figure 5B).

Moreover, ISG15 mRNA expression was enhanced when Selgantolimod and Motolimod were combined with RLR agonist Poly(I:C) (LMW)/Lyovec compared to stimulation with the agonists alone, with a 1,5–2 fold increase in gene expression being observed 8 h after stimulation (Figure 4B).

Both Selgantolimod and Motolimod (TLR8) were able to induce MxA, with upregulation in gene-expression observed following stimulation compared to the medium control. Following co-stimulation expression increased further, resulting in a 1,5 fold increase. This increase proved significant for the combination of Motolimod and Poly(I:C) (LMW)/Lyovec. Telratolimod alone did not induce MxA, compared to the medium control, and Poly(I:C) (LMW)/Lyovec alone induced low levels. Co-stimulation of Telratolimod with Poly(I:C) (LMW)/Lyovec induced high levels of MxA, however, due to variation between donors no significance could be obtained (Figure 4C).

IP10, A3G, OAS1, Trim22 and Trim5α were also investigated, however, with the exception of mild synergy following co-stimulation with Selgantolimod (TLR8) and Poly(I:C) (LMW)/Lyovec for IP10 and A3G, no relevant increases in ISG expression was found compared to the medium control. Furthermore, with the exception of IP10 for Vesatolimod, no synergy was observed between TLR7 and RLR agonists (Supplementary Figures S2A–E, S3A–D).

## Discussion

Induction of effective anti-HIV-1 immune responses are thought to be vital in developing an HIV cure. TLR7 agonists have been investigated for their potency in contributing to HIV cure. Vesatolimod (GS-9620), a selective TLR7 agonist, was able to induce antiviral responses in PLWH undergoing ART (9). Furthermore, in non-human primates viral rebound was delayed after treatment with TLR7 agonist and cessation of ART (24). Thus, there is a need for efficient inducers of antiviral immunity in HIV-cure strategies. Stimulation *via* TLR and RLRs are important parts of immune sensing and activation. While the individual pathways have been extensively studied little is known of crosstalk between various systems. We therefore investigated several immunomodulatory agents for their potency alone or in combination to activate and induce antiviral immune responses. All TLR-agonists we tested had the potency to activate both DCs and PBMCs. Our data suggest that TLR8 triggering in DCs and PBMCs is more potent than TLR7 and, importantly, that crosstalk with RLR strongly modulates immune responses leading to enhanced pro-inflammatory cytokine IL-12p70 and type I IFN responses. The differences observed following triggering of TLR8 versus TLR7 might be due to low expression of TLR7 in DCs and PBMCs, and that TLR8 induces NFκB more efficiently than TLR7 (43, 44). Thus, targeting both TLR and RLR pathways could be effective to induce potent antiviral immunity in HIV cure strategies.

We observed a dose-dependent response for all TLR-agonists, with TLR8 agonists reaching a plateau at lower concentrations concerning IL-6 production and IL-12 responses. Interestingly, TLR7 agonists Vesatolimod and Gardiquimod were more potent than R837, suggesting that agonist structures are also important in efficient triggering of TLR7, which could provide insights in the failures observed when using TLR7 agonists as a LRA (12). Utilizing a stronger immune inductor, such as TLR8 agonists, could provide better results.

The pro-inflammatory cytokine IL-12p70 is important for induction of adaptive immunity to viruses by inducing T helper type 1 differentiation, as well as activation of NK cells and cytotoxic T cells that are required for effective antiviral responses (45). Although some TLR8 agonists induced IL-12p70 alone, co-stimulation of both TLR7/TLR8 and TLR8 agonists with PolyI:C/Lyovec enhanced IL-12p70 expression in DCs and PBMCs at multiple concentrations.

Type I IFN responses are important in inducing innate antiviral immunity such as restriction factors APOBEC3G or TRIM5a (46–49). Moreover, type I IFN responses are crucial in the induction of antiviral immune response such as induction of Th1 and follicular Th and cytotoxic T cell responses (48, 50–53). Interestingly, although TLR agonists alone were able to induce type I IFN responses, we observed that co-stimulation of TLR8 and RLR enhanced type I IFN responses. Co-stimulation with a Poly(I:C) (LMW)/Lyovec induced higher levels of ISGs IL-27A mRNA as well as ISG-15 mRNA, with a similar effect observed when observing secreted IL-27 and IFN-β. This effect was observed for both TLR8 agonists, Selgantolimod and Motolimod and, while not statistically significant, a similar trend was observed for Telratolimod, a TLR7&8 agonist.

These data suggest that TLR8 signaling is modified by RLR signaling probably at the level of IFNR signaling. Previously we have shown that IFNR signaling induced by IFN-*β* intersects with RLR signaling at the level of IKKε phosphorylation, leading to ISGF3 formation and induction of IL-27A mRNA transcription (27). TLR8 agonists alone also indued IL-27A expression, which could be crosstalk between IFNR and TLR8 signaling as TLR8 also induced low levels of IFN-β in DCs. Strikingly, when looking at fully formed IL-27, TLR8 agonists performed comparably to the medium controls, suggesting abrogation of IL-27 formation following previous transcription. RLR signaling lead to the formation of IL-27 as also described by Sprokholt et al. Subsequently, added TLR8 signaling providing a potent increase in cytokine secretion, for both IFN-β and IL-27.

These data suggest that co-stimulation of TLR8 with RLR pathway might enhance antiviral immunity *via* induction of type I IFN responses as well as IL-12p70. However, T helper cell differentiation as well as CD8 T cell activation studies are required to further understand the importance of crosstalk between these pathways, especially since TL8 and RLR activation decreased IL-6 expression.

IL-12 is observed in successful ART regimens with persistent CD8^+^ T-cell activation (54). This suggests a restriction of IL-6, with induction of IL-12 may be beneficial when forming competent immune responses. These observed effects may provide next steps in reactivation and elimination of the HIV-1 reservoir and will be the topic of future research.

To summarize, our findings underscore the importance of crosstalk between various antiviral sensing systems in creating durable and robust immune responses.

## Data availability statement

The original contributions presented in the study are included in the article/supplementary materials, further inquiries can be directed to the corresponding author/s. Raw data will be made available upon reasonable request.

## Ethics statement

Ethical review and approval was not required for the study on human participants in accordance with the local legislation and institutional requirements. Written informed consent for participation was not required for this study in accordance with the national legislation and the institutional requirements.

## Author contributions

KV and TG conceived and designed experiments. KV, KW, and TK performed the experiments. KV and KW acquired data and analyzed data. KV, MN, NK, GB, and TG interpreted data and contributed to scientific discussion. KV, GB, and TG wrote the manuscript with input from all listed authors. TG perceived of the original study idea and was involved in all aspects of the study. TG vouches for the completeness and accuracy of the data. All authors contributed to the article and approved the submitted version.

## Funding

Funding for this study was obtained through a ZonMW/Aidsfonds; NL4Cure: Bridging shock and kill strategies (446002508).

## Conflict of interest

The authors declare that the research was conducted in the absence of any commercial or financial relationships that could be construed as a potential conflict of interest.

## Publisher’s note

All claims expressed in this article are solely those of the authors and do not necessarily represent those of their affiliated organizations, or those of the publisher, the editors and the reviewers. Any product that may be evaluated in this article, or claim that may be made by its manufacturer, is not guaranteed or endorsed by the publisher.
