# Supplementary figures and images for: Crosstalk between TLR8 and RIG-I-like receptors enhances antiviral immune responses
Source: Front Med (Lausanne). 2023 May 16;10:1146457. doi: 10.3389/fmed.2023.1146457 (PMC10227620; doi:10.3389/fmed.2023.1146457)

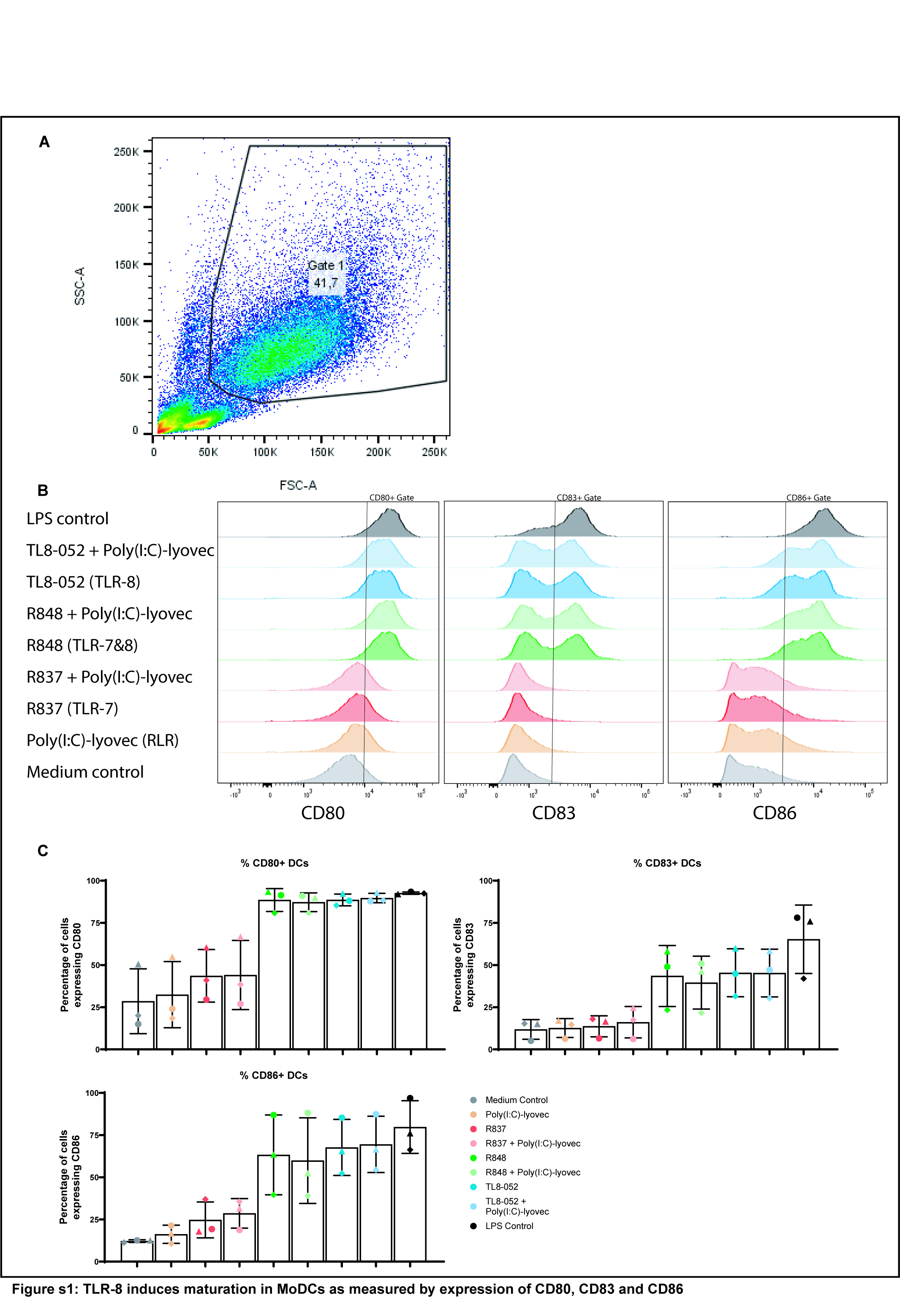

Supplement: Supplementary file 1 [file Image_1.tif]

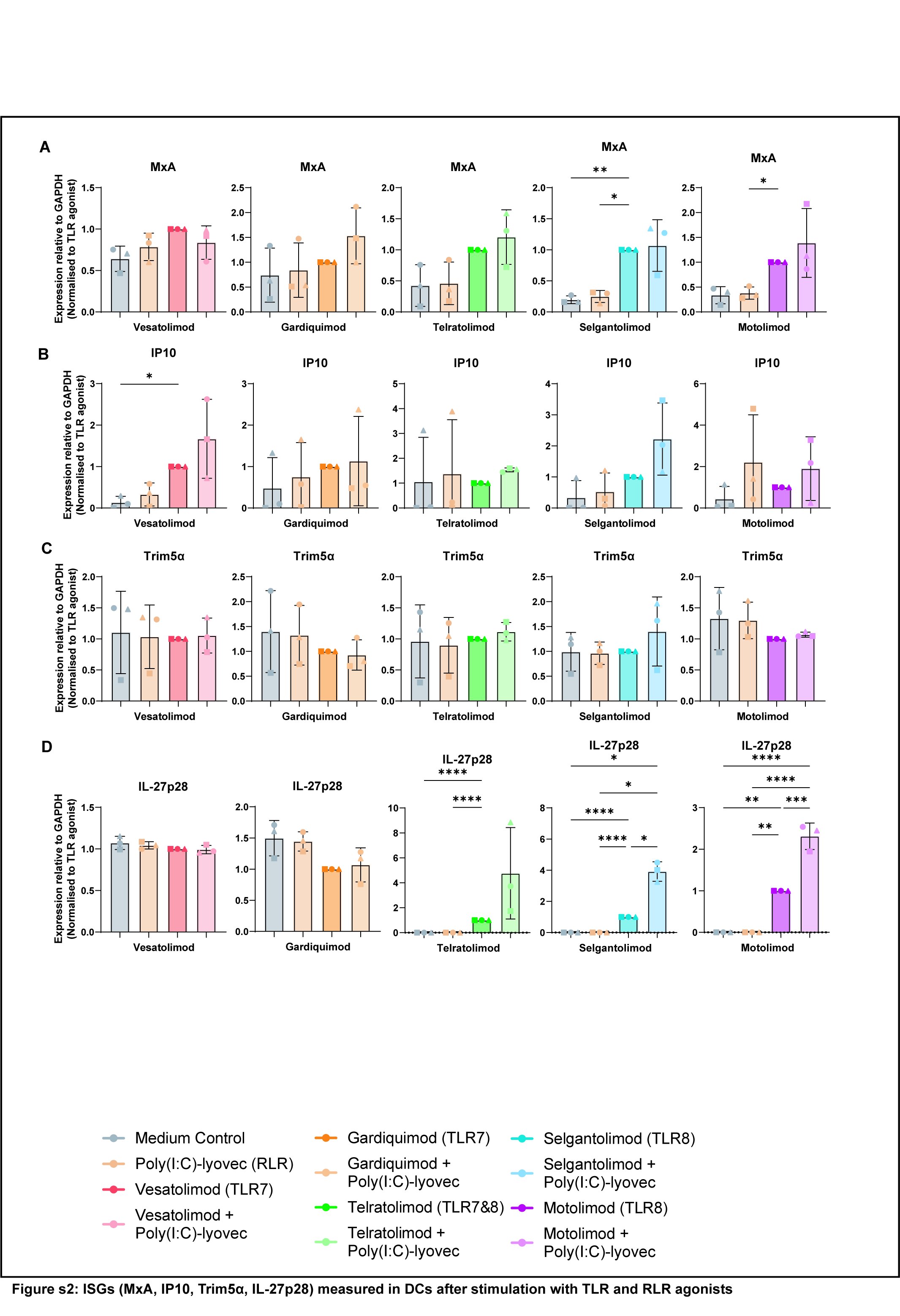

Supplement: Supplementary file 2 [file Image_2.tif]

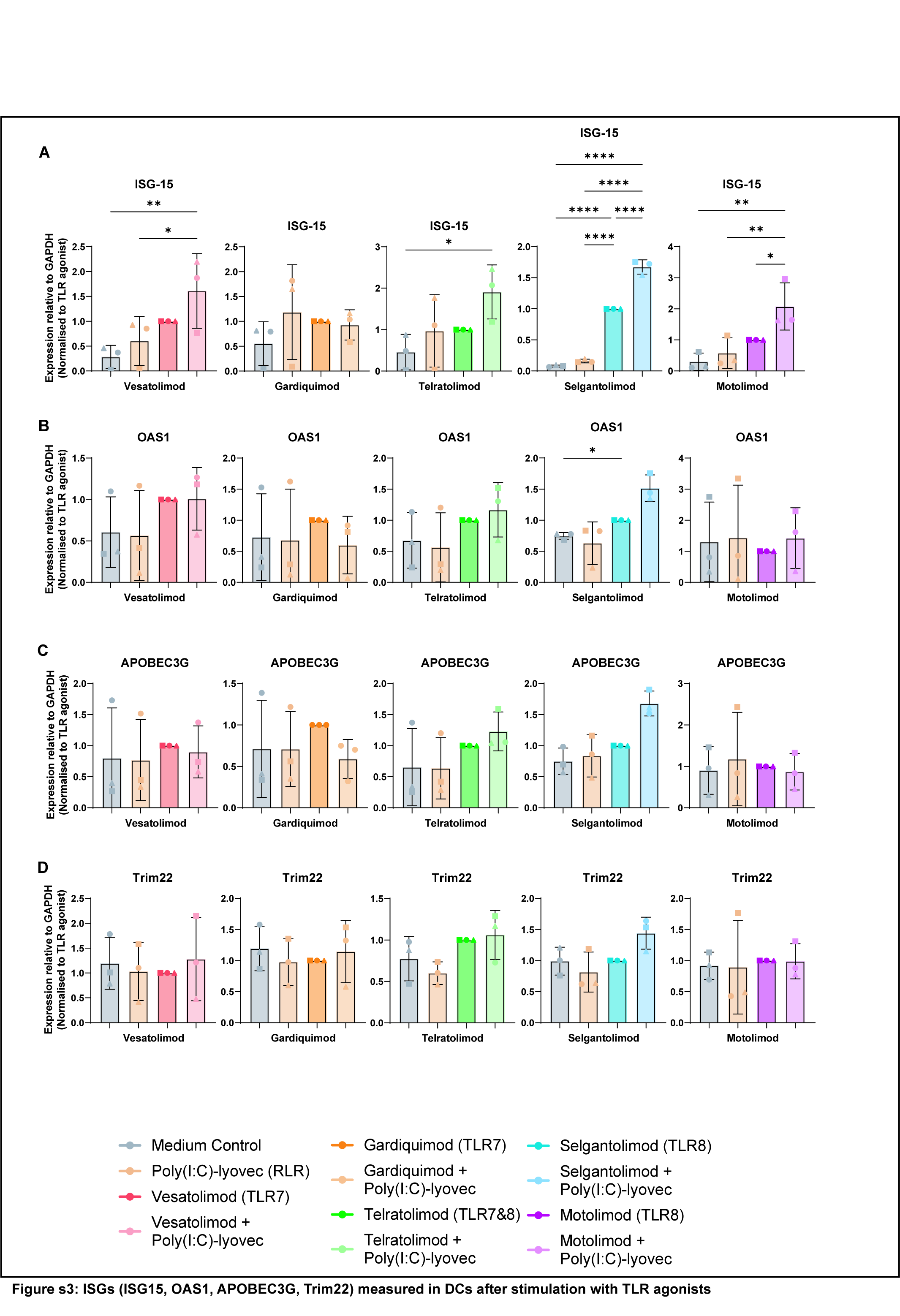

Supplement: Supplementary file 3 [file Image_3.tif]

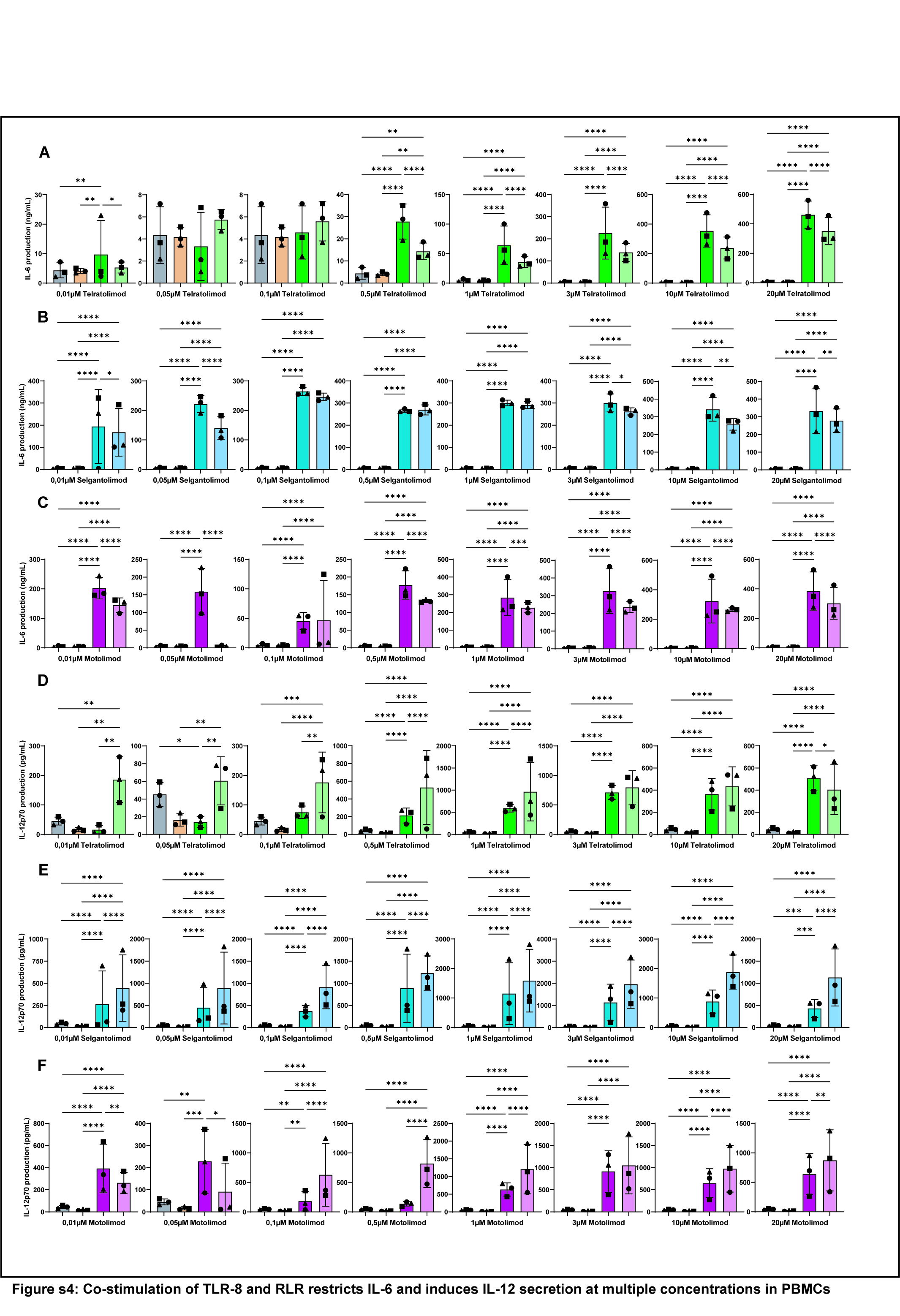

Supplement: Supplementary file 4 [file Image_4.TIF]
